# Resilient sustainable current and emerging technologies for foodborne pathogen detection

**DOI:** 10.1039/d4fb00192c

**Published:** 2024-09-05

**Authors:** Debarati Bhowmik, Jonathan James Stanely Rickard, Raz Jelinek, Pola Goldberg Oppenheimer

**Affiliations:** a School of Chemical Engineering, University of Birmingham Birmingham B15 2TT UK p.goldbergoppenheimer@bham.ac.uk; b Department of Physics, Cavendish Laboratory, University of Cambridge Cambridge UK; c Department of Chemistry, Ben Gurion University of the Negev 84105 Beer Sheva Israel; d Healthcare Technologies Institute Mindelsohn Way Birmingham B15 2TH UK

## Abstract

Foodborne pathogens such as *Salmonella*, *Escherichia coli* and *Listeria* pose significant risks to human health. The World Health Organization estimates that 2.2 million deaths per year are directly caused by foodborne and waterborne bacterial diseases worldwide. Accordingly, detecting pathogens in food is essential to ensure that our food is safe. This review explores the critical role of novel technologies in enhancing food safety practices whilst delving into adopting and integrating innovative, resilient and sustainable approaches in the food supply chain. Further, applying novel, emerging advanced analytical techniques such as Raman spectroscopy and nanotechnology based biosensors in food contamination detection is discussed. These advanced technologies show the promise of real-time monitoring, traceability, and predictive analytics to identify and mitigate potential hazards before they reach consumers. They can provide rapid and accurate results and ensure the integrity of food products. Furthermore, the herein-highlighted synergistic integration of these technologies offers a promising path toward a safer and more transparent food system, thereby addressing the challenges of today's globalised food market and laying the platform for developing multimodal technologies for affordable, sensitive and rapid pathogen detection along the different stages of the food chain, from “farm to fork”.

## Abstract 2

Integration of industry 5.0 principles: human-centric, sustainable, resilient with the “farm-to-fork” supply chain *via* using advanced technologies to ensure food safety and quality whilst linking sustainable practices with socioeconomic innovation.

## Graphical abstract

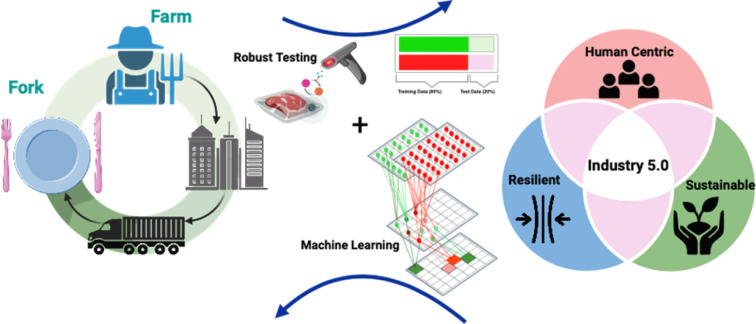

**Sustainability spotlight**

The ability to detect pathogens in food at different stages of the food supply-chain is paramount to ensure that the food we eat is safe. We overview recent sensing developments and look to the future of novel diagnostics to enhance food integrity whilst integrating resilient and sustainable approaches in the food supply-chain. This review fills a critical gap in current and emerging pathogen sensing techniques, combining exciting breakthroughs in molecular sciences and engineering, carving-out ambitious but undoubtedly desired capabilities for real-time monitoring, traceability and predictive analytics, identifying and mitigating potential hazards *prior to* reaching consumers. The synergistic integration of such sensors lays the platform toward safer, more transparent food systems, addressing the challenges of globalised food markets *via* multimodal, rapid pathogen detection from “farm-to-fork”.

## Introduction

The global food supply and safety are threatened due to the rising population, limited Earth resources and other external factors, sustainability, and climate change. Bacteriotoxins and mycotoxin residues present a considerable threat to food safety, as their complete removal during food processing is difficult.^1^ Among these, Ochratoxin A, Patulin, Fumonisins and Aflatoxin B1 (AFB1) are especially concerning, as they are widely recognized as the most dangerous ones of the aflatoxins, primarily due to their high mutagenic and carcinogenic potential.^2^ Thus, producing safe foods to high standards is essential to retain security and public trust. Ensuring food safety protects public health, reduces the economic and social burden of food-borne diseases, and contributes to overall economic growth.^3^ Yu *et al.* (2023) developed a microelectromechanical system (MEMS) to support photothermal immunoassay for detecting harmful mycotoxins like Aflatoxin B1 (AFB1) in food, offering point-of-care testing that is crucial for ensuring food safety, especially in regions with limited resources.^4^

The agriculture and aquaculture industries are the major contributors to the economy of a developing country. 80% of the harvested food is consumed by humans, and the remaining 20% is for livestock.^5^ Rapid changes in the climate have threatened food resources and driven the food industry to move towards sustainable systems. The agri-and aqua-food industries are moving towards more sustainable systems where food quality is strictly monitored. Despite following conventional agriculture and aquaculture practices, the food industry suffers from numerous pathogen infections. One-third of the food produced globally is lost or wasted due to microbial attacks. Approximately 931 million tons of global food wastage were recorded in 2021 by the United Nations Programme.^6^ Food waste due to microbial spoilage can happen at any stage along the food supply chain, including during production, processing, transport at the point of sale and during consumption.^7^ Food microbial contamination across the global food chain is the primary reason for spreading infectious pathogens from farm to fork. According to the Food and Agriculture Organization of the United Nations (FAO), climate changes have majorly impacted the survival and growth of pathogens in both the environment and the food matrix, resulting in increased food spoilage and subsequently affecting human health.^7^

There are 2.4 million cases of food poisoning in the UK each year, costing up to £9 billion.^8^ This impacts both economies and healthcare systems globally. Furthermore, the increasing concerns around climate change, land and water use and loss of biodiversity have encouraged more resilient technology to retain food for increasing time without compromising food security and quality.^9^ A resilient system is defined as “the system's capacity to be robust, adaptable, and versatile to change to maintain the desired state of food security and safety in the long term whilst also favouring clean economic growth that mitigates our impact on the environment”.^10^ Globalisation has triggered growing consumer demand for a variety of foods, resulting in complex and longer global food chains (WHO, 2022). International trade in food and agriculture has doubled between 1995 and 2018 to US $1.5 trillion due to lower trade barriers along global food chains. Therefore, prioritising the reduction of food loss and waste is critical for the transition to sustainable agrifood systems that improve the efficient use of natural resources, lessen their impact on the planet and ensure food security and nutrition.^11^ This network of international food trade has now renewed focus on food safety standards as higher volumes of perishable goods move across borders around the world.^12^

Due to urbanisation and habitat loss, animals are now living in closer proximity to humans, which enables rapid transmission of zoonotic pathogens to humans from animals and food vectors.^13^ Meanwhile, in the food-producing and processing industry, it is crucial to identify starter cultures, probiotics, and food-borne pathogens to prevent any batch failures, and spoilage or to generally monitor hygiene in the production facility.^14^ Longer testing times and the need for specialised equipment expertise mean delayed releases of products, which leads to the food having a reduced shelf life. Thus, timely detection and identification of these pathogens is important to prevent their spread locally, nationally, and internationally to minimise and prevent the distribution of contaminated batches of food to avoid an international crisis and ensure food safety and quality from farm to fork.^12,14,15^ Also, allowing faster product release cycles can save businesses millions of dollars and provide them with a clear competitive edge.^14,15^ Food safety is a growing international priority to protect human health, the environment, and economies. Due to the urgent need to reform food security to reduce both commercial losses and burden on health care systems many global organizations such as the World Health Organization (WHO), FAO, United Nations Environment Programme (UNEP), International Association for Food Protection (IAFP), World Resources Institute (WRI), World Food Programme (WFP) and International Food Information Council (IFIC) closely work together to ensure food safety along the food chain from production to consumption.

Post Brexit, Britain has been struggling to cope with food regulations due to a shortage of staff and toxicologists to monitor food and chemical safety. The Food Standard Agency (FSA) has lost full access to the EU's Rapid Alert System on Food and Feed (RASFF), which provides member states with information on food safety incidents, increasing the time and effort required to manage food safety incidents. Additionally, the Health and Safety Executive (HSE) ceases to have access to the chemical safety data underpinning the EU's Registration, Evaluation, Authorisation and Restriction of Chemicals (REACH) regulations. It is estimated that it will cost £800 million (US $901 million) to replicate these data in the UK REACH system.^16^ Thus, the modernisation of food process control, together with rapid microbiological testing, can help bring improvement in food security.

Microbial spoilage and infection of food results from Gram-positive and Gram-negative bacteria, members of the fungi and mould families.^7,17,18^ Several genera of bacteria, such as *Bacillus* spp. and lactic acid bacteria (LAB), are responsible for the spoilage of fruits, vegetables, meat and dairy products.^19^ Food safety and security are inevitably interlinked.^20^ Foodborne diseases hinder socioeconomic development by burdening healthcare systems and harming national economies, tourism and trade,^21^ whereas food spoilage and waste create a vicious cycle of malnutrition and hunger. Both food-borne illness and food spoilage impact global food security and food safety. Noteworthily, there is an overlap between food spoilage organisms and food pathogens. Rarely, foodborne pathogens are associated with food deterioration. However, it may be possible to have food spoilage organisms cause foodborne illnesses, also known as “pathogenic spoilage organisms”. Thus, it is necessary to prevent spoilage to reduce the risk of foodborne illness.^22–24^

**Fig. 1:**
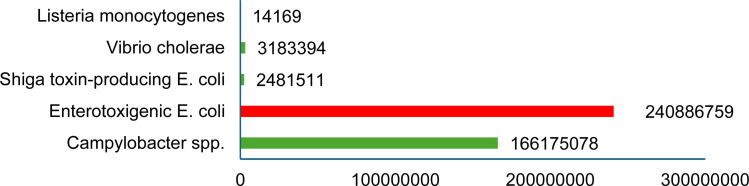
The burden of foodborne diseases worldwide, 2024 (World Health Organization, 2024).

Strategies aimed at reducing waste and resource footprints are crucial for sustainability, food security and safety. Intensifying food production and creating a circular food system could be key to achieving these goals.^25^ Zoonotic pathogens transmitted through food are a serious public health issue and result in substantial economic losses worldwide.^26^ The majority of foodborne diseases are caused by eating raw meat and dairy products infected with pathogenic microorganisms. Pathogenic microorganisms such as *Campylobacter*, *Salmonella*, *Escherichia coli* O157:H7, *Listeria*, *Vibrio* spp., and *Streptococcus* spp. are primarily responsible for foodborne disease outbreaks in humans, farmed animals, and harvested crops (Table 1).^26^ It has been reported that *Listeria*-related poisoning has a mortality rate of almost 16%.^28^ Most foodborne illnesses are characterised by acute diarrhoea accompanied by stomach cramps, headache and fever.^29^ Campylobacteriosis is caused by *Campylobacter jejuni* and *Campylobacter coli*.^29^ Occasionally, *Campylobacter jejuni* infections may trigger Guillain-Barre Syndrome (GBS) which causes peripheral neurological injury in humans previously infected with *Campylobacter* spp.^30^ (Fig. 1). In 2021, 30 EU/EEA countries have reported 129 960 confirmed cases of campylobacteriosis.^29^ In the UK, a recent outbreak of *Listeria* and *Salmonella* had been reported and many foods such as cheese and chocolate have been recalled by Müller, Ferrero and Cadbury.^31–33^ Other organisms which impact biosecurity are nanobacteria, prions, noroviruses and fungal toxins that have been studied relatively poorly.^34^ Recently, in 2022 outbreaks of Hepatitis A virus (HAV) genotype IB had been reported in the UK, six European countries along with Arizona, California, Minnesota, and North Dakota in the USA through the consumption of fresh organic strawberries.^35,36^

**Table 1:** Pathogenic spoilage and foodborne microorganisms

Organism	Types of food spoiled	Types of spoilage
*Bacillus* spp.	Bread, minced meat, fish, kebabs, pizza	Slime^19^
	Potatoes	Rotting
	Dairy products	Biofilms, curdling
*Lactobacillus* spp.	Bread	Ropy,^27^ slime
*Lactococcus* spp.	Yogurt	
*Pediococcus* spp.	Cheese	
*Enterococcus* spp.		
*Streptococcus* spp.		

The genus *Bacillus* includes species such as *Bacillus subtilis*, *Bacillus cereus*, and *Bacillus licheniformis*, which are widely distributed in both terrestrial and marine ecosystems. *Bacillus* species are rod-shaped microorganisms with the ability to generate heat-resistant spores under adverse conditions. These spores can survive elevated temperature treatment and can spoil dairy and bakery products reducing the shelf life.^37^ Therefore, *Bacillus* species are recognised as a causative agent of food poisoning and spoilage. Members of the *Bacillus* group are reported to produce extracellular toxins which in most cases can cause an allergic reaction in humans.^38^*Bacillus subtilis* may also produce an extracellular toxin known as subtilisin,^38^*Bacillus licheniformis* produces lichenysin A, *Bacillus pumilus* produces pumilacidin, *Bacillus mojavensis* and *Bacillus subtilis* produces surfactin,^39^*Bacillus amyloliquefaciens produces* amylosin and *Bacillus cereus* produces cereulide toxin.^40^ These are heat stable lipopeptides, which are formed during the vegetative growth in stored food thus, if present in food it is resistant to destruction by heating.^41^*Bacillus subtilis*, *Bacillus cereus*, and *Bacillus licheniformis* have been widely reported for ‘ropy bread’.^42^ Among others, *Bacillus cereus* can also spoil pasteurised milk, meat and fish^43^ and *Bacillus stearothermophilus* can spoil canned foods.^38^

Lactic acid bacteria include the genera *Lactococcus*, *Lactobacillus*, *Pediococcus*, *Enterococcus* and *Streptococcus*.^44^ They are best known as fermentative agents in meat, vegetables, and dairy products to produce salami, sauerkraut and cheese. However, spoiling occurs when these bacteria grow in certain foods, such as luncheon meats, vegetable salads and liquid milk.^45^ Ready-to-eat (RTE) refrigerated meat products, when packed under aerobic conditions, can be spoiled by lactic acid bacteria. Spoilage of meat is associated with ropy slime formation, generation of offensive metabolites for example histidine and ornithine, turbidity, discoloration and odour development. The major biochemical changes in meat occur when LAB metabolises the sugars, nucleotides, peptides and amino acids in the meat. The metabolic by-products produce compounds such as organic acids (acetic acid, lactic acid, propionic acid^46^), hydrogen sulphide (H_2_S), ammonia (NH_3_), indole, skatole, acetylene vinyl and 3-methylbutanol,^46^ biogenic amines such as cadaverine, histamine, putrescine, spermine and tyramine.^47^*Lactobacillus* and *Pediococcus* are also reported to be major spoilage organisms in the brewery industry.^46^ The appearance and taste of alcoholic beverages are affected. They produce turbidity, buttery odour and sourness which makes them unpalatable due to the formation of diacetic acid, lactic acid and extracellular polysaccharide.^46,48^

*Shewanella* spp. is a Gram-negative bacteria known to be the dominant spoilage organism of sea food.^49^*Shewanella* (formerly known as *Pseudomonas putrefaciens* and *Alteromonas putrefaciens*) belongs to the Shewanellaceae family and is found in both fresh and marine water ecosystems.^49,50^*Shewanella* spp. have the potential to adhere to fish mucus and form strong biofilms with high amine metabolic ability.^49,50^ This pathogen even at low temperatures can metabolise trimethylamine-*N*-oxide (TMAO) to trimethylamine (TMA) or degrade nitrogenous substances resulting in deterioration in fish quality.^50^ Elevated levels of biogenic amines like histamine, cadaverine and putrescine, while reduced concentrations of spermine and spermidine, are indicators of fish spoilage.^51^ According to reports, the most common cases of amine food poisoning are linked to the consumption of mackerel fish.^49^

While the need for innovative detection technologies is evident. Before exploring the cutting-edge detection technologies, it's crucial to understand the conventional methods that have long underpinned food safety practices. The next section will review these traditional approaches, highlighting their strengths and limitations, setting the stage for a discussion on how modern innovations are enhancing and, in some cases, replacing these traditional practices.

### Conventional methods for the detection of spoilage

When facing foodborne illness outbreaks, rapid and facile screening strategies need to be employed at the point of need (PoN)/point of care (PoC) to monitor and prevent epidemics.^52^ Early detection and diagnosis are crucial in planning treatment and subsequent prevention strategies.^53^ This is especially useful in detecting outbreaks that occur over large geographic areas.^54^ The response time between exposure and confirmed diagnosis can vary greatly depending on the type of infection and the monitoring method employed. Thus, rapid, accurate, simple and portable diagnostic tests are required to enable faster diagnosis, direct medical interventions, identification of the best control method and mitigation of the transmission of infectious diseases. Conventional methods, including immunoassays, DNA/RNA analysis, and other nanomolecular technologies, have significantly improved reaction time, sensitivity, and ability to discriminate between and within species. Additionally, recent electrochemical and optical signal transduction advances have made it easier to integrate cutting-edge assays into useful miniaturised devices.

The merits and demerits of conventional culture-based methods, as well as molecular (PCR) and immunological (ELISA) (Fig. 3A) methods for pathogen detection, are summarized in Table 3. While these methods offer significant advantages, they also present challenges in accuracy and specificity, as detailed in the table.

Regardless of these drawbacks, the majority of food and dairy industries employ fluorescence flow cytometry (Fig. 3B) and fluorescence-based real-time PCR (Fig. 3C). Automated commercial PCR-based kits such as RapidFinder™, ThermoFisher Scientific (Table 2) and food safety kits employ a variety of antigens to detect contamination and the presence of many GMO products for authentication for 30 samples in 2.5 hours. The N-Light Listeria Rapid Test^56^ by Nemis Technologies, founded in 2018, employed chemiluminescence to detect *Listeria* spps. on working surfaces. Other commercially available instruments such as Bactobox by SBT Instruments^57^ (Fig. 5E) and BactoScan™ FC+ by Foss Analytics^58^ (Fig. 5G), have employed fluorescence flow cytometry to enumerate bacteria. They are commercially used by various food safety testing laboratories and industries across the globe. However, these non-invasive fluorescent techniques suffer from various disadvantages, such as photobleaching, reduced depth penetration, and the broad-spectrum fluorophores limiting the number of reporters that can be detected simultaneously in an assay, also known as a multiplexed assay. Other limitations include low sensitivity for detecting microbial contaminants, and autofluorescence from pathogens can affect the signal and lead to false positives. Besides, good laboratory practices (GLP) are required for sample preparation and interpretation of results. Thus, these protocols are confined to the laboratory space and require trained staff. This enables new opportunities for developing sensitive, portable, resilient, and sustainable analytical devices for detecting food-borne pathogens. Thus, the compromise between the complexity and the accuracy of the colourimetric and fluorescence assays has laid the platform for Raman spectroscopy as a potential analytical tool for detecting food-borne and spoilage pathogens.

**Table 2:** Commercial detection methods for major foodborne pathogens

Organism	Method	Technique	Manufacturer
*Bacillus* spp.	Duopath^®^ enterotoxins	Lateral flow device phenotyping profile	Merck kGaA
	*Bacillus cereus* ID		Microgen
	Phenotype Microarray™		Bioproducts Ltd
			Biolog Inc.
*Lactobacillus* spp.	API®	Biochemical testing	bioMérieux
S*hewanella* spp. *E. coli* O157:H7	DrySpot™	Latex agglutination test	ThermoFisher scientific
	VIP® gold EHEC	Lateral flow test	Merck
	TRASIA I® AG singlepath®	ELISA immunochromatographic rapid test based on gold-labeled antibodies	Merck
			Merck
			Merck
	RapidFinder™	PCR	ThermoFisher Scientific
	MicroSEQ	PCR	Applied Biosciences
*Listeria* spp.	Singlepath^®^	Lateral flow test	Merck
	VIP^®^ gold Listeria	Lateral flow test	Merck
	SureTect™	PCR	ThermoFisher Scientific
*E.coli*, *Lactococcus lactis*, *Pseudomonas fluorescens*	Bactobox^®^	Flow cytometry	SBT instruments
Microbial cells	BactoScan™ FC/FC+	Flow cytometry	FOSS

**Table 3:** Overview of pathogen detection technologies: advantages, disadvantages and recent innovations

Technology	Advantages	Disadvantages	New modifications and applications
Culture-based methods	- Gold standard for pathogen detection, the international organization for standardisation (ISO), food and drug administration (FDA) and food safety and standards authority of India (FSSAI)	- Time-consuming (days to weeks)	- Automated culture systems: reduced time for detection and lower labour requirements
	- High accuracy in identifying viable organisms	- Labor-intensive	- Microfluidic culture chips: enhanced sensitivity for low-level contamination and improved speed of culture development
		- Impractical for perishable food with short shelf life	
		- Requires skilled personnel	
PCR (polymerase chain reaction)	- High sensitivity and specificity	- Requires precise primer design	- Digital PCR (dPCR): increased sensitivity and precision in quantification
	- Can detect a wide range of pathogens	- Potential for false negatives due to inhibitors or non-specific amplification	- Multiplex PCR: reduced cost and time for multiplex testing
	- Relatively quick (few hours)	- Contamination risk	- Nanoparticle-enhanced PCR (biomimetic magnetic nanoparticles (BMNPs): enhanced detection in complex matrices due to nanoparticle use^55^)
	- Capable of detecting low levels of contamination	- Expensive equipment	Isothermal amplification (*e.g.*, LAMP, SEA)
		- Requires trained personnel	- Recombinase polymerase amplification (RPA): increased speed and simplicity for field use
		-False positive or negative results-DNase or RNase activity	- CRISPR-based isothermal amplification: higher specificity with reduced off-target effects
			- SEA-LAMP: combines advantages for better performance
ELISA (enzyme-linked Immunosorbent assay)	- Widely used and accepted	- Dependent on antibody specificity	- Magnetic bead-based ELISA: improved sensitivity and speed
	- Relatively quick and easy to perform	- Risk of cross-reactivity leading to false positives/negatives	- Nano-ELISA: enhanced performance for low-abundance targets with the use of nanomaterials
	- Cost-effective for large sample volumes	- Limited to known pathogens	- Automated ELISA platforms: higher throughput with automated systems
	- High sensitivity and specificity when using well-characterized antibodies	- Requires lab-based equipment and trained personnel	- Quantum dot-based sensors: increased signal stability and sensitivity
	- Can be multiplexed for detecting multiple targets	Fluorescence-based ELISA assays – issues with photobleaching and autofluorescence	- Time-resolved fluorescence: reduced false positives due to background noise
			- FRET-based assays: enhanced ability to monitor interactions in real-time
Next-generation sequencing (NGS)	- Comprehensive detection of known and unknown pathogens	- Expensive and time-consuming	- Long-read sequencing (*e.g.*, oxford nanopore): better detection of structural variations and complex regions
	- High throughput	- Requires high-quality DNA/RNA extraction	- Single-cell sequencing: insights into the heterogeneity of microbial communities and rare variants
	- High resolution for epidemiological tracking and surveillance	- Complex data analysis and interpretation	-Whole genome sequencing: comprehensive genetic analysis
	- Comprehensive genetic analysis (*e.g.*, antimicrobial resistance, virulence factors)	- Unsuitable for rapid, PoC diagnostics	- Metagenomics: comprehensive profiling of microbiomes
	- Provides extensive data on antimicrobial resistance and virulence	- Resource intensive with long turnaround times	- Real-time sequencing: rapid identification and response during foodborne outbreak
		- Complex bioinformatics expertise and significant infrastructure	
Biosensors	- Rapid detection and real-time monitoring	- Stability issues (*e.g.*, reagent-dependent biosensors)	Electrochemical biosensors-graphene-based electrodes, enzyme-free electrochemical sensors, 3D-printed electrodes, nanomaterial based biosensors- graphene, au, ag, Al based electrodes and/or paper-based, wearable sensors: detection of ultra-low concentrations of pathogens in complex matrices, affordable and portable for widespread use
	- Portable and potentially field-deployable	- Calibration and validation challenges	- AI-driven analysis for integrated lab-on-a chip: enhanced accuracy and ease of use in remote and resource-limited settings
	- High sensitivity and specificity with appropriate design	- Often requires expensive and specialized materials (*e.g.*, nanoparticles)	
	- Label-free detection possible	- Potential for false positives due to non-specific binding	
	- High sensitivity and selectivity	- Complex fabrication and calibration	
	- Fast response time (minutes)		
	- Compatible with various biochemical assays		
Raman spectroscopy	- Non-invasive and label-free	- Low sensitivity for low-concentration analytes without enhancement (*e.g.*, SERS)	- Portable Raman devices, surface-enhanced Raman spectroscopy (SERS) with machine learning integration- high sensitivity even at low concentrations, enhanced data analysis for complex samples, portable
	- High specificity due to unique spectral fingerprints	- Expensive instrumentation	- Plasmonic nanostructures: higher sensitivity and selectivity
	- Portable versions available for field use	- Limited by interference from complex food matrices	- Microfluidic-SERS: faster and more efficient processing for small sample volumes
	- Fast detection (minutes)		
	- Can be used for multiplexing		

### Nucleic acid based assays

Nucleic acid sequencers at PoC have been in demand for screening infectious and antimicrobial-resistant pathogens for quality assurance in the food industry. Nucleic acid based assays detect specific DNA or RNA sequences in target pathogens.^59^

PCR amplification and electrophoresis-based profiling are commonly used in gene sequencing. Different techniques like Restriction fragment length polymorphism (RFLP-PCR), Amplified Fragment Length Polymorphism (AFLP), and Randomly Amplified Polymorphic DNA (RAPD-PCR), which target specific gene sequences, have been developed to identify foodborne pathogens.^60^ The RAPD technique can identify and differentiate genetic clusters of various isolates of the same pathogen.^61^ This technique can be exploited to differentiate pathogenic and non-pathogenic strains.^60,61^ To achieve the desired results, enzymatic cleavage of DNA using restriction enzymes followed by targeted amplification of the resulting fragments with specific PCR primers and subsequent separation of the PCR products is involved.^62^ Strand Exchange Amplification (SEA) was developed to overcome the drawbacks of conventional PCR. SEA is an isothermal amplification method in which the 16s rRNA gene of the pathogen is targeted.^63,64^ This highly sensitive assay can detect 0.1 to 0.01 pM of genomic DNA in an hour. The use of short-length amplicons in SEA facilitates its application in lateral-flow strips. SEA-based lateral flow strips have been developed to detect *Salmonella* spp. with a sensitivity of 6 Colony Forming Unit (CFU) per mL.^65,66^ The developed lateral flow assay produced results in a timely manner within thirty minutes. Additionally, the results of SEA correlate well with conventional PCR.^67^ Both isothermal technologies and PCR share common drawbacks, notably the generation of non-specific amplification products caused by primer dimers. This occurrence can occur when primers bind to non-target nucleic acids or when one primer is directly copied onto another.^68^ This non-specific amplification can lead to reduced yield, sensitivity, or specificity due to the inefficient use of assay components and the occurrence of non-specific exponential amplification.^68^

Fluorescence *in situ* hybridisation (FISH) has also been utilised for the detection of infectious pathogens using species-specific probes that bind to the ribosomes of the pathogens. Fluorescence intensity can be visualised when the short fluorescence-labelled DNA probes or nucleic acid-mimicking peptides (PNA) hybridise the target sequence. FISH has a shorter time of detection compared to PCR.^69^ Various food-borne pathogens have been detected with high specificity using FISH using a PNA probe.^69^ This technique can provide unique information regarding special resolution, morphology and differentiation of key pathogens in a mixed sample. The oligonucleotide-probe FISH combined with a direct viable count allows the differentiation of live and dead cells.

Loop-mediated isothermal amplification (LAMP) has been utilised in significant research.^70^ The isothermal LAMP technology is an exception to other isothermal technologies as LAMP utilises a specific set of primers, usually from four to six, and a unique DNA polymerase. Other isothermal technologies such as Hybridization Chain Reaction (HCR) techniques have been used to amplify the signal for the detection of biomarkers. HCR is enzyme-free and suitable for applications where enzyme stability is a concern, while LAMP is enzyme-dependent and offers rapid amplification under isothermal conditions. Zhang *et al.* (2020) presented a highly sensitive photoelectrochemical (PEC) biosensor for detecting carcinoembryonic antigen (CEA) using g-C_3_N_4_/CuInS_2_ nanohybrids and CoOOH nanosheets, coupled with the hybridization chain reaction (HCR) and etching reaction. This innovative approach offered a low detection limit of 5.2 pg mL^−1^, demonstrating significant potential for early cancer diagnosis and broad applications in both medical diagnostics and food safety.^71^

*Bacillus stearothermophilus* (Bst LF) or *Geobacillus* spp. (GspDNA) polymerase is used for its high strand displacement activity.^65^ At constant temperature, unique polymerase amplifies the target gene sequence and accumulates double-stranded DNA.^65^ Despite LAMP's sensitivity and specificity, the technique still suffers from non-specific amplification primarily caused by numerous large primers that serve as polymerase substrates, resulting in false-positive results^68^ (Fig. 4). Gavrilov *et al.* (2022) designed an isothermal amplification method SHARP (SSBHelicase Assisted Rapid PCR). This technique is centred on a modified helicase and single stranded DNA binding protein (SSB) combined with standard PCR reagents which can produce amplicons with lengths of up to 6000 base pairs.^72^ Colorimetric LAMP (Loop Mediated Isothermal Amplification) provides rapid and sensitive detection of foodborne pathogens, *V. parahaemolyticus* and *V. cholerae* in sea food samples. Integrating molecular beacons containing HRPzyme sequences (horseradish peroxidase mimicking DNAzyme) and complementary oligonucleotides enhances the specificity and sensitivity of these assays.^73^ The detection sensitivity of this assay is reported to be in the range of 9–50 CFU per mL with an assay time of sixty minutes. However, the results are more sensitive for the isothermal SEA assay. Molecular beacon-based colourimetric LAMP is an ultra-sensitive detection technology that avoids yielding any false-positive results commonly associated with colourimetric LAMP using hydroxyl naphthol blue.^74^

Rolling Circle Amplification (RCA) and nucleic acid sequence-based amplification (NASBA) are also isothermal nucleic acid amplification methods which operate at lower temperatures than LAMP. RCA can rapidly synthesise multiple circular RNA or DNA molecules in plasmids and viruses. Zhang *et al.* (2018) presented a highly sensitive bio-bar-code-based photoelectrochemical immunoassay for detecting prostate-specific antigen (PSA) using rolling circle amplification and enzymatic biocatalytic precipitation. This innovative approach achieved a low detection limit and high specificity, demonstrating significant potential for practical applications in medical diagnostics and potentially adaptable for food safety testing.^75^ An RCA-based microfluidic device coupled with a thin film photodiode fluorescence readout has a limit of detection (LOD) of 0.5 fM, enabling the detection of viral RNA with only a 5 μl sample at 37 °C, offering a robust solution for detecting foodborne viruses.^76^

DNA chips or microarrays have been recently used in studies to differentiate foodborne pathogens by identifying virulence genes.^77^ DNA or RNA probes bound to a surface have been used for the parallel detection of pathogens. This technique is highly specific and has high throughput but still encounters the challenge of cross-hybridization. An on-chip PCR technique was further developed to overcome a multi-step protocol for the microarray-based analysis. The one-step on-chip PCR technique involves amplifying DNA samples in the liquid phase and semi-nested solid phase PCR on the surface of coated glass slides. During the thermal cycling, a PCR product is generated in the fluid phase, which serves as a template for the solid phase primer extension. Elongated solid-phase immobilised products are then subject to second-strand synthesis, initiating a solid-phase PCR driven by the immobilised nested primer and the second primer in solution. The glass chip contains hundreds of covalently attached specific oligonucleotides designed to interrogate multiple single nucleotide positions within the genomic regions of interest. The amplified PCR products can then be visualised, analysed, and interpreted using computer-automated software to identify a positive signal of the fluorescent dyes incorporated into the amplicons during PCR. On-chip PCR requires little hands-on time since it integrates amplifiers and genotypes into a single assay. Only a single pipetting step is needed to launch the reaction from a pre-prepared master mix. On-chip PCR can efficiently detect conserved genes, such as the 23S rRNA genes, using consensus primers,^78^ thus, allowing simultaneous analysis of a high number of sequence loci.

Hence, point-of-care molecular diagnostics require simple and rapid technologies and methods to meet the ASSURED (affordable, sensitive, specific, user-friendly, rapid, robust, equipment-free, and deliverable to end-users) criteria developed by the WHO.^79^ Thus, further advancements in reducing the total reaction time and cost for sustainable techniques for PoC diagnostics have been developing. For instance, Lee *et al.* (2020) developed a novel nanoplasmonic on-chip PCR (npPCR) for rapid precision molecular diagnostics using a white LED.^78^ The nanoplasmonic pillar arrays (NPA) efficiently induce plasmonic photothermal heating on the glass substrate, resulting in highly enhanced light absorption over the visible spectrum. The NPA effectively, rapidly, and precisely controls the temperature of the PCR due to the ultrafast energy conversion and heat dissipation of the Au nanoislands. This npPCR allows rapid thermal cycling of 30 cycles for 3 minutes and 30 seconds between 60 °C and 98 °C using a simple LED light. In addition, the wafer-level fabrication of NPA enables low-cost and disposable PCR chips for PoC diagnosis. Therefore, the ultrafast amplification of λ-DNA (0.1 ng μl^−1^) and rapid detection of infectious diseases using complementary DNA (cDNA) (0.1 ng μl^−1^) offers a promising solution for the detection of viral food-borne pathogens.

The CRISPR/Cas systems, known for their role in genome editing, can precisely recognise and cut specific DNA and RNA sequences. This ability makes them suitable for multiplexing, allowing a single diagnostic test to identify multiple targets with high sensitivity, reaching concentrations as low as attomolar (10^−18^ mol l^−1^) target molecules.^80^ Zeng *et al.* (2022) developed a portable photoelectrochemical (PEC) biosensor for the point-of-care detection of human papillomavirus-16 (HPV-16) using a CRISPR-Cas12a system. The biosensor employs a hollow In_2_O_3_–In_2_S_3_-modified screen-printed electrode (SPE) as the photoactive material. The CRISPR-Cas12a system specifically recognizes and cleaves G-quadruplex structures, which are involved in biocatalytic precipitation, leading to changes in photocurrent that can be measured using a smartphone-based device. This system demonstrated high sensitivity, with a detection range from 5.0 to 5000 pM and a detection limit as low as 1.2 pM. The integration of the PEC biosensor with a smartphone enables rapid, low-cost, and portable diagnostics, making it suitable for use in remote and resource-limited settings.^81^

Similarly, Gong *et al.* (2022) developed a PEC biosensing platform for the detection of microRNA-21 (miR-21), a key biomarker for various diseases including cancer. The platform integrates a catalytic hairpin assembly (CHA) for target amplification and reduced graphene oxide-anchored Bi_2_WO_6_ (rGO-BWO) as the photoactive material. The CHA reaction produces double-stranded DNA (dsDNA) that activates the trans-cleavage activity of Cas12a, leading to the release of alkaline phosphatase (ALP) from magnetic beads. The ALP catalyzes the formation of ascorbic acid (AA), which enhances the photocurrent response of the rGO-BWO-modified electrode. The biosensor demonstrated a detection limit of 0.47 fM for miR-21, with excellent stability, selectivity, and reproducibility.^82^ While microRNA-21 (miR-21) itself is not currently recognised as a biomarker for foodborne diseases, the presence of certain pathogens or toxins in food can lead to changes in host gene expression, including alterations in specific microRNAs and can serve as indicators of infection or inflammation caused by foodborne pathogens. Thus, the concept of utilising microRNAs as biomarkers in food safety is still in the exploratory stages. Future research could investigate whether miR-21, or other specific microRNAs, are involved in the host's response to foodborne pathogens and could be used as indicators of such diseases where innovative approaches such as PEC sensing can be employed.

Gao *et al.* (2021) proposed a novel nucleic acid detection methodology, “PCF” (PCR-CRISPR-Fluorescence based nucleic acid detection), for the sensitive detection of bacteria. PCF is a CRISPR Cas13a (CRISPR is Clustered Regularly Interspaced Short Palindromic Repeats) based system for bacteria detection. In this approach, the target DNA is first amplified through PCR and then transcribed into RNA using T7 transcriptase. The resulting RNA activates the RNase activity of the Cas13a protein, enabling specific and efficient RNA-based detection and manipulation. The activated Cas13a protein cleaves the quenched fluorescent probe to generate a fluorescent signal. The PCF detection methodology exhibited excellent sensitivity capable of detecting *Salmonella* genomic DNA with a minimum of 101aM or 10 CFU mL^−1^ of *Salmonella* bacteria in 2 hours. The methodology demonstrated good specificity with no cross reaction with other common foodborne bacteria.^83^

Matrix-assisted laser desorption/ionization-time of flight mass spectrometry (MALDITOF MS) has been widely used as a reference method for directly identifying microorganisms by analysis of biopolymers such as DNA, RNA, proteins, peptides and carbohydrates. It is also used to sense and detect the presence of antigens, biomarkers of antimicrobial resistance, which has revolutionised pathogen identification. Nevertheless, further work is required to refine research protocols and ensure their validation for regular practical use of this technology.^84,85^ Li *et al.* (2022) further proposed a novel approach to mass tag-mediated surface engineering for multiplexed detection of bacteria using MALDI-TOF MS. Here, the bacteria were detected in three main steps: target bacteria capture using aptamer, signal amplification by rolling circle amplification (RCA), and mass tag detection using MALDI-TOF MS. This strategy claims to have overcome the dependence on microbial mass spectral databases for the identification of target bacteria^86^ and foresees its application in milk safety monitoring.^84–86^

### Whole genome sequencing

The above-discussed technologies require prior information on the target pathogens for designing probes for assays. Conversely, Next-Generation Sequencing (NGS) has been proposed as an alternative to DNA or RNA assays, which allows surveillance and detection of all DNA or RNA-based food-borne pathogens and uncultured microorganisms. Although sequencing is heavily dependent on the quality of the extracted DNA library, the growing insights into pathogen genomics and pathogenesis have made NGS an interesting tool for identifying food-borne pathogens and controlling disease outbreaks.^87–89^ Whole Genome Sequencing (WGS) of the pathogens provides information about genetic manipulation, mutational bias, geographical pattern bias, antimicrobial resistance, virulence pattern, allelic variants and genes responsible for the presence of toxins to investigators.^66,90^ WGS as a molecular tool is rapidly becoming economically feasible. WGS can inform risk assessments and thereby help shape food policy. Metagenome Assembled Genomes (MAGs) combined with genome annotation allows taxonomic profiling with higher resolution and facilitate identification at the species level. Genomic-level investigation of the functional potential of microorganisms provides insight into unknown species. Commercial companies like Eurofins' DNA Patho Tracker and SGS provide services to track contamination using WGS. However, a successful transition to a wider use of WGS would necessitate investments in infrastructure and training for correctly interpreting data, trends, noise and random variation. However public–private partnerships and robust policy frameworks must be structured to govern the technology's unforeseen consequences.^36,90,91^

### Biosensors

The increasing demand for rapid and accurate detection of pathogens has motivated the development of innovative biosensors exploiting the use of signal-enhancement molecules and particles. Biosensors are “analytical devices” designed to detect single molecular entities in a complex matrix to produce detectable physical changes. Among these, Photoelectrochemical (PEC) biosensors as highly sensitive analytical devices that detect single molecular entities in complex matrices by converting photo-induced electron transfer events into measurable electrical signals. These biosensors consist of components like excitation light sources, electrochemical workstations, and signal-receiving devices and influence the photoelectric properties of materials to achieve high sensitivity and specificity.^92^ Yu *et al.* (2024) describe a portable immunobiosensor for detecting cardiac troponin I (cTnI) using antibodies and CuS–Pt nanofragments. The biosensor converts the biological recognition of cTnI into a measurable physical change through photothermal and pressure-based mechanisms, providing a low-cost, detector-free visual readout suitable for point-of-care testing.^93^ In contrast to nucleic acid-based assays or immunoassays, an ideal biosensor is reagent less and does not change composition as a consequence of making the measurement. Relevant studies by Lu *et al.* (2023) and Zeng *et al.* (2023) have highlighted cation exchange (CE) or photothermal and polarity-switchable photoelectrochemical (PEC) immunoassays.^94,95^ Together, these innovations highlight the potential of biosensors to provide accurate, reliable measurements while remaining stable and reagent-free, embodying the key features of an ideal biosensor.

Most biosensors incorporate a naturally derived macromolecule, like an enzyme or an antibody, identifying a specific physical signal unique to the targeted molecule and further developing a detector tailored specifically for that setup. For example, Xu *et al.* (2022), have utilised aptamers to specifically bind to prostate specific antigen (PSA), initiating a hybridisation chain reaction (HCR) that amplifies the signal by forming double-stranded DNA on magnetic beads. This process facilitates the assembly of alkaline phosphatase (ALP) enzymes, which hydrolyse sodium thiophosphate to produce H2S, enhancing PEC performance and creating a visible colour change for PSA detection.^96^ Similarly, Yu *et al.* (2022) employed a classical sandwiched immunoreaction model, where the target cardiac troponin I (cTnI) is captured by a monoclonal anti-cTnI capture antibody (mAb1) and detected by a monoclonal anti-cTnI detection antibody (mAb2) conjugated with glucose oxidase (GOx). This triggers a cascade of enzymatic reaction, producing a photothermal signal that induces a colour change in the thermochromic paper for visual detection.^97^

A variety of nanoparticles have been used to develop biosensors for the detection of bacterial pathogens. Metal nanoparticles coupled with antibodies, oligonucleotide probes and enzymes have been exploited in fluorescence assays and colourimetry pathogen sensors. For example, self-aggregating gold nanoparticles (AuNPs) conjugated with oligonucleotides have been used in colourimetric sensors. Unique properties of nanoparticles, such as their surface plasmon resonance (SPR) and enzyme-mimic catalytic activity, have paved the way for rapid and sensitive surface-enhanced Raman scattering (SERS), microfluidic devices, and electrochemical biosensors. Biosensors have gained significant interest in bacterial pathogen detection due to their easy handling, rapid response time, cost-effectiveness, and high sensitivity.

### Fluorescence biosensors

Fluorescence-based biosensors have been an attractive tool for probing protein interactions and viewing conformational changes in complex biological matrices. For instance, the development of a highly sensitive, interference-resistant fluorescence immunoassay for carcinoembryonic antigen (CEA) detection has been reported by Qiu *et al.* (2017) and Lv *et al.* (2018). Qiu *et al.* (2017) developed a paper-based analytical device (PAD) utilising CdTe/CdSe quantum dots (QDs), where fluorescence quenching is triggered by glucose release from DNA-gated mesoporous silica nanocontainers, achieving a detection limit of 6.7 pg mL^−1^.^98^ Similarly, Lv *et al.* (2018) employed PAD using NH_2_-MIL-125(Ti), which undergoes a structural change in response to wet NH_3_ generated through a sandwich immunoassay with gold nanoparticles, enabling detection with a limit of 0.041 ng mL^−1^.^98,99^ Fluorescence detection is widely used for real-time tracking in a complex environment, assessing the status of a specific target, highlighting cellular interactions and monitoring disease progression. Nanozymes and nanocatalysts offer a promising alternative to natural enzymes for constructing novel nanomaterial based fluorescent ELISA biosensors-“nano-ELISA”.^100^ A variety of enzyme-mimic nanocatalysts, nanozymes such as metal oxide (nanoparticles) NPs, carbon-based nanomaterials, noble metal NPs, metal–organic frameworks (MOFs), and plasmonic Cu_2−*x*_SySe_1−*y*_ nanocatalysts, have been successfully developed for fluorescence-based biosensors. Advantages include easier preparation, lower cost, and higher catalytic stability under harsh conditions.^101^ Recently, click chemistry has gained significant popularity for studying biological processes by efficient fluorescent labelling of reporter molecules. The CNN (copper containing nanocatalyst) clickase-based immunoassay exhibited high analytical performances for the quantification of *Salmonella enteritidis* in the linear range of 10^2^–10^6^ CFU mL^−1^ with a limit of detection as low as 11 CFU mL^−1^.^102^ N-Light, Nemis Technologies, a commercial brand, has employed chemiluminescence to detect *Listeria* spps. on the food industry working surfaces using a swabbing technique.^56^

Gold nanoparticles (AuNPs) exhibit interesting optical properties and can enhance or reduce the fluorescence intensity of fluorescent dyes. AuNPs have been exploited for biosensing of *Pseudomonas aeruginosa* with a detection limit of about 60 cells and a detection time of 10 minutes.^103^ Bi *et al.* (2020) developed an AuNP-based dual sensor system of colourimetric and fluorescence responses for the detection of biogenic amines such as histamine as spoilage indicator of seafood, with a LOD of 0.1 μM (ref. 104) (Fig. 3E). Similarly, novel immunosensors based on reduced graphene oxide have been developed to detect *E. coli* with a lower limit of detection (LOD) of 10 colony forming units (CFU) ml^−1^ achieved with a detection time of 30 minutes.^105^ Due to their smaller size, higher sensitivity and specificity, nanoparticles have also been exploited in electrochemical biosensors.

### Electrochemical biosensors

Electrochemical biosensors offer a label-free, rapid and sensitive detection of foodborne pathogens. These sensors measure the changes in current, resistance or voltage by redox or antigen-binding reactions^106,107^ (Fig. 3F). They can be classified into conductometric, amperometric, and potentiometric sensors. The electrochemical biosensors mimic various biochemical assays, thus making them highly compatible for detecting food-borne pathogens in a fast-paced and demanding environment of food safety monitoring. For example, a silica nanoparticle-based electrochemical biosensor has been developed to detect *E. coli* within 5 to 30 minutes with an LOD of 10^3^ CFU mL^−1^. Since silica-based nanoparticles could lead to false positive results, an electrochemical sensor based on nanocomposites of polyaniline (PANI) with gold nanoparticles (AuNPs) and MoS^2^ for the detection of bacterial pathogens had been developed by Raj *et al.* (2021). A self-assembled monolayer of mercaptopropionic acid on AuNPs was introduced to covalently immobilise the antibodies to prevent the non-specific adsorption of the target pathogen on the electrode surface.^108^ Thus, a hybrid nanocomposite of organic PANI and inorganic components (MoS^2^ AuNPs) offered excellent electroactivity for bacterial pathogen detection with high selectivity and sensitivity with a low limit of detection of 10 CFU mL^−1^ and detected *E. coli* within 30 minutes.^108^ Another study developed a bio-nanocomposite-modified pencil graphite electrode (PGE) using polypyrrole (PPy)/AuNP/multiwalled carbon nanotubes (MWCNTs)/chitosan (Chi). This hybrid bio-nanocomposite platform was immobilised with *E. coli* O157:H7 monoclonal antibodies and reported selectivity to *E. coli* O157:H7 with a LOD of 30 CFU mL^−1^ in PBS buffer.^107^ Stratford *et al.* (2019) demonstrated the rapid detection of bacteria by electrically inducing membrane potential dynamics on the bacterial cell membrane. It is possible to differentiate and quantify live and dead bacteria using this technology.^109^ This rapid detection capability is crucial for minimizing the risk of contamination and ensuring the safety of food products throughout the supply chain.

The potentiometric transducing technique has led to the development of electronic noses (e-nose) and electronic tongues (e-tongue). This technique involves the detection of electrical potential due to the generation of chemical species on the electrode surface. The biochemical reaction occurs on the working electrode surface, and the variation in the potential between the reference and working electrode is recorded. E-noses are semi-selective gas sensors, which interact with volatile molecules, producing physical or chemical reactions and sending a signal to a computational device that employs Pattern Recognition Methods (PARC). E-noses utilise various types of gas sensors, including metal oxide semiconductors (MOSs), quartz crystal microbalances (QCMs), and surface acoustic waves (SAWs).^110^ Quartz Crystal Microbalance (QCM) sensors have been used to study and detect the deterioration of milk due to bacterial contamination with *Pseudomonas fragi* and *Escherichia coli*.^111^ In another study, an electronic nose composed of metal oxide semiconductor field-effect transistors (MOSFET) and Taguchi sensors (TGS) to detect and monitor the growth of spoilage bacteria in milk.^112^ Shauloff *et al.* (2021) developed a novel artificial nose based on interdigitated electrodes (IDEs) coated with carbon dots (C-dots). Bacteria-specific volatile molecules induce capacitance changes on adsorbed electrode-deposited C-dots, which can discriminate amongst bacteria through “volatile compound fingerprint” for bacterial species^113^. Similarly, E-tongues use various non-specific chemical sensors with high stability and cross sensitivity. E-tongues have been used to monitor and assess the quality of food.^114^ It has been reported that gold electrodes could differentiate *Salmonella enterica* and *Klebsiella pneumoniae*.

Additionally, the gold electrode could discriminate decreasing concentrations of *Escherichia coli*, from 1 × 06 CFU mL^−1^ to 1 × 10^−2^ CFU mL^−1^ in pasteurised milk.^111,115^ Ghrissi *et al.* (2021) developed a lab-made potentiometric E-tongue multisensory device using a lipid polymeric membrane coupled with two cylindrical arrays and an Ag/AgCl reference electrode to establish the potentiometric fingerprints of four different food-contaminating microorganisms, including two Gram-positive (*Enterococcus faecalis*, *Staphylococcus aureus*) and two Gram-negative (*Escherichia coli*, *Pseudomonas aeruginosa*). This unique lipid membrane senses the electrostatic interactions and hydrogen bonds with hydroxyl, amine, and carbonyl groups of the bacterial outer membrane.^114^ Other potentiometric devices such as ISFET (ion-sensitive field-effect transistor), FET (field effect transistor) and MOSFET (Metal Oxide Semiconductor Field-Effect Transistor) have been widely used for rapid detection of Gram-positive bacteria. FET detectors based on hybrid nanostructures of MoS_2_/TiO_2_ conjugated with Vancomycin have demonstrated high selectivity towards Gram-positive bacteria due to their preferential binding with the terminal d-alanyl-d-alanine of the peptidoglycan layer of Gram-positive bacteria. This biosensor showed high sensitivity for *Staphylococcus aureus* with 10^2^ CFU mL^−1^.^116^

Moreover, the integration of electrochemical biosensors with portable devices, such as smartphones, is a game-changer for food safety monitoring. One such smartphone-based electrochemical biosensor, iEAT, has been developed for the detection of on-site food allergens. The biosensor consists of a smartphone app, an electronic reader, and a disposable allergen extraction device. The allergens are collected *via* immunomagnetic enrichment and chromogenic electron mediators such as horseradish peroxidases have been used to generate electrochemical signals, which are then processed by the microcontroller unit (MCU), and results are displayed on a mini display screen. The device comes with a Bluetooth communication module, a rechargeable battery, and a card-edge connector to insert an electrode board.^117^ Such tools enable immediate detection and response to food safety hazards, which is particularly valuable in environments where quick decision-making is essential, such as in food production facilities, distribution centres and retail outlets.

### Raman spectroscopy in the food industry

**Fig. 2:**
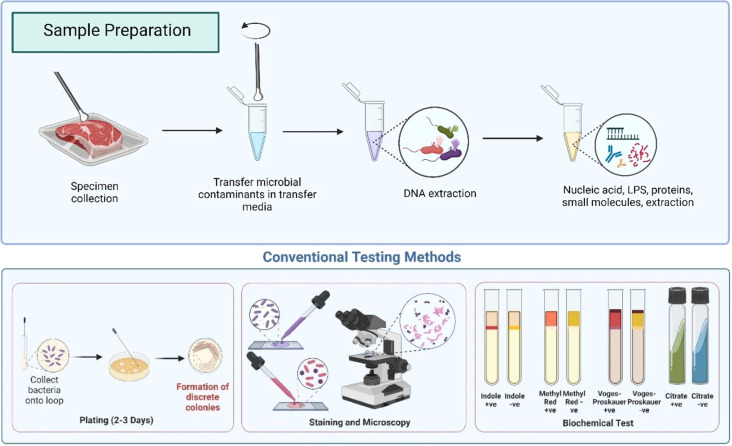
Overview of the conventional testing methodology in microbiology. Created with Biorender.

Conventional methods of detecting foodborne pathogens primarily utilise biochemical markers and thus heavily depend on phenotypic features and metabolic reactions, which may produce false negative results (Fig. 2). As discussed above, genomic analysis and serological analysis (nucleic acid-based assays) can detect pathogens with relatively high accuracy though they suffer lengthy sample preparation. Additionally, some pathogens are difficult to grow in laboratory settings, thus leading to delayed diagnosis, which often results in severe losses and poses a risk to food security. To overcome delays in microbial testing, resilient, robust, reproducible, and sustainable identification of microorganisms needs to be recognised in modern microbiological laboratories to avoid food security threats. Over the preceding years, non-invasive optical spectroscopy techniques such as Raman spectroscopy, infrared spectroscopy such as IR Biotyper®, Bruker^118^ and hyperspectral imaging have gained extensive popularity as an analytical method for the quick characterisation and identification of microorganisms.

Raman spectroscopy, in particular, is highly advantageous for biological applications due to its minimal background noise in aqueous samples, low cost, convenience of use, quick analysis, and extensive information on the chemical makeup, structure, and interactions of biomolecules in microorganisms. Raman spectroscopy is a type of vibrational spectroscopy involving the study of the interaction of monochromatic radiation with molecular bond vibrations, providing information about characteristic fundamental vibrations which are then employed for structural elucidation. From the perspective of the quantum theory of radiation, Raman spectroscopy is based on inelastic light scattering of electromagnetic waves (UV, visible and IR) upon collision with matter.^119^ UV-Resonance Raman Spectroscopy (UVRRS) is a powerful technique where the molecules are illuminated with two laser excitation wavelengths: a resonating UV wavelength light (200–514 nm) and a non-resonating wavelength (785–1064 nm) to produce results of resonance with UV Raman excitation.^120^ UVRRS produces a highly selective Raman effect for biological targets containing aromatic rings and amide groups such as proteins and DNA, which otherwise typically show fluorescence when using conventional Raman Spectroscopy.^121^ The unique “vibrational fingerprints” provide chemical information on the biological samples. Raman spectra of the microorganisms reflect their entire molecular make-up, which includes both species-specific components (such as carotenoids) and a wide variety of vibrational modes of taxonomic biomarkers such as DNA/RNA, proteins, lipids, and carbohydrates. Raman spectra, thus, reveal both the genetic and phenotypic signatures of the microorganism referred to as spectral fingerprints. The composition of the proteome, cell wall or lipopolysaccharide (LPS) structure varies amongst different organisms. For example, the teichoic or teichuronic acid units differ in amino acids and sugar units in different groups and classes of bacteria, thereby exhibiting species-specific Raman spectral fingerprints. This method can also detect within minutes, revealing the biochemical difference between samples and enabling the characterisation, discrimination, and identification of bacteria at species and subspecies levels.^14^

**Fig. 3:**
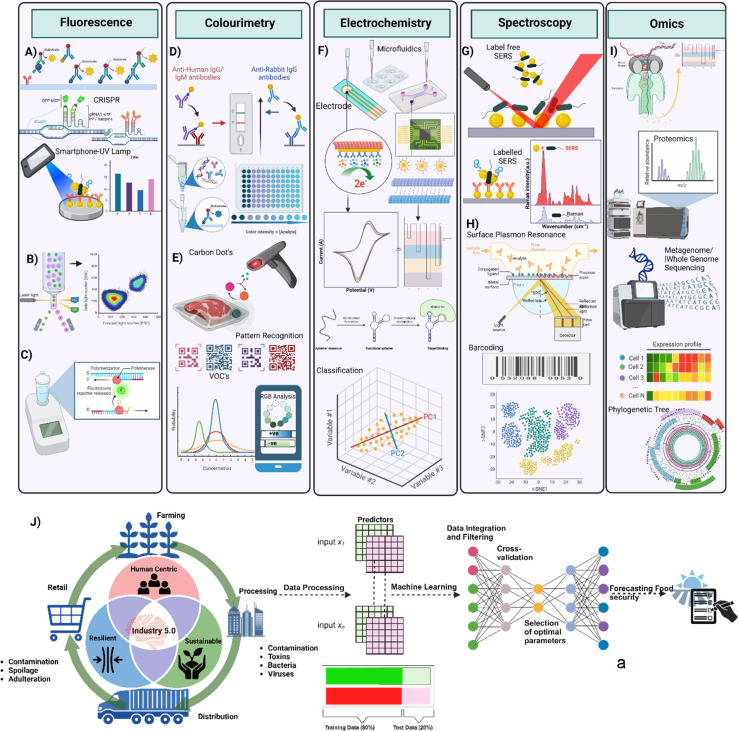
(A) ELISA, CRISPR-based fluorescence assay for nucleic acid detection, and smartphone-UV lamp integration for data visualisation, (B) flow cytometry for single-cell analysis using laser-induced fluorescence, (C) fluorescent-based isothermal amplification for rapid DNA detection, (D) lateral flow immunoassays for detecting human and rabbit IgG/IgM antibodies using colorimetric signals, (E) detection of volatile organic compounds (VOCs) with carbon dot sensors and pattern recognition, (F) microfluidics-based electrochemical sensing for detecting biomolecules with redox reactions and current–potential measurements, (G) surface-enhanced Raman spectroscopy (SERS) for label-free and labelled detection of molecules, (H) surface plasmon resonance (SPR) for real-time biomolecular interaction analysis and barcode-based sample classification, (I) high-throughput techniques like proteomics, metagenomics, and whole-genome sequencing for comprehensive analysis of cellular expression profiles and phylogenetic relationships, and (J) industry 5.0's integration of human-centric, resilient, and sustainable practices with AI and data-driven decision-making for optimized manufacturing and supply chain management. Created with Biorender.

Moreover, conventional Raman spectroscopy is limited by a weak Raman scattering signal where a small fraction of the incident photons is scattered inelastically. Raman spectroscopy may fail to detect low concentrations of biomarkers, thus leading to the development of SERS (Surface Enhanced Raman Scattering) (Fig. 3G). This technology provides high sensitivity by using nanometal structures to enhance the low-concentration single molecule of Raman signal by several magnitudes, typically 10^7^ to 10^14^. This enhancement is due to the localised surface plasmons (LSPs) on the surface of the metallic nanostructure. The electromagnetic enhancement of the LSP depends on the size, geometry, shape of the nanostructures, charge transfer mechanism and surface interaction of the analyte molecule. Due to these observed enhancements, SERS has been used to identify and detect biomarkers with high sensitivity, accuracy and multiplexing in the food industry. The accuracy of SERS is attributed to the narrow bandwidth of Raman peaks, which facilitates their clear extraction from the background signal, thereby reducing false positives that could arise from varying background noise. The narrow Raman bandwidth also allows the simultaneous detection of multiple peaks associated with different biomarkers, enabling multiplexing and enhancing diagnostic accuracy by correlating multiple spectral peaks with specific conditions. These features render Raman spectroscopy particularly advantageous for non-invasive and point-of-care (PoC) applications.

**Fig. 4:**
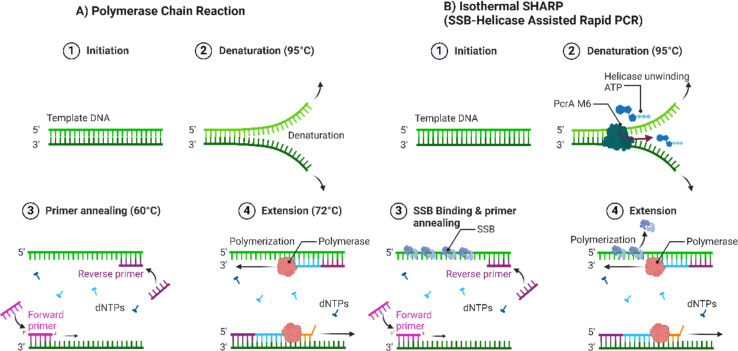
PCR *versus* SHARP. (A) PCR consists of alternating cycles of heating and cooling temperatures involving denaturation at 98 °C, annealing at 45–60 °C and extension at 72 °C. Using Taq polymerase 1 (*Thermus aquaticus*). (B) SHARP is an isothermal DNA amplification reaction. It uses an engineered helicase PcrA M6 which has ×4 greater unwinding activity and SSB (single stranded DNA binding protein) to unwind the DNA helix and enable primer binding. Bst-LF (*Bacillus stearothermophilus* DNA polymerase 1) is used for DNA strand replication. Adapted from Gavrilov *et al.* 2022.^72^ Created with Biorender.

### SERS as advanced optical biosensors

Recently, Au and Ag nanoparticles as SERS substrates have been used to detect chemical residues of acephate (organophosphate), carbendazim (benzimidazole fungicide), thiamethoxam (neonicotinoid) and tricyclazole (fungicide) in basmati rice and the surface of fruit peels.^122^ Identification and detection of foodborne pathogens in food by SERS have posed difficulties due to the interference of food components with the signal of the bacteria. In addition, the bacterial membrane protein and carbohydrate structures vary at different phases of their life cycle, posing difficulty in identifying species of bacteria. Despite this, SERS for the identification of bacteria in food has been demonstrated. In most cases, using SERS for the detection of bacteria involves the isolation of the bacterial strains, followed by utilising Raman spectroscopy for identification, thus, making the sample preparation more tedious and time-consuming. Although the detection technique employed is rapid, the sample preparation steps could be complex and labour-intensive, which poses a significant challenge in a commercial setting where speed and efficiency are crucial. However, pure pathogenic bacterial cultures have revealed phenotypic species-specific fingerprints of carbohydrates, nucleic acids, lipopolysaccharides, nucleic acids, peptidoglycans, quinones, cytochromes, phospholipids and proteins present in the bacterial membrane and some endogenous biomolecules.

To date, two principal methods of bacterial detection have been developed using SERS biosensors. They are label-free and label-based strategies (Fig. 3G). The label based methods are further grouped as (a) antibody-based, (b) aptamer-based, and (c) nucleic acid-based SERS biosensors. These SERS biosensors utilise antibodies, aptamers, and nucleic acid as biorecognition elements to capture target molecules and use SERS as readout. The receptors capture the pathogens and pathogen-released proteins, antibodies, and DNA/RNA of the target microorganism. The label-based SERS sensor is sensitive and allows multiplexed detection with different Raman reporter molecules. Although this method is sensitive, it is more complicated and expensive and exhibits stability issues. Compared to microbiological and molecular methods, biosensors offer immediate and accurate pathogen detection, aiding in assessing food contamination levels.^123^ Yang *et al.* (2022) reported the use of Ag nanostructure SERS for the detection of ss-DNA (stx2) in Shiga toxin-producing *Escherichia coli* with a limit of detection 0.4900 aM ss-DNA (stx2). The substrate was coated with thiol-ssDNA and 6-mercapto-1-hexanol (HS(CH_2_)_6_OH) for bonding target ss-DNA and blocking Ag nanostructure, respectively.^124^

The label-free method directly detects the original Raman signal of bacterial cells or bacterial metabolites attached to the SERS substrate or the SERS active NP substrate in a solution.^125,126^ This method is most used for pathogen identification by processing the intrinsic vibrational fingerprints of the pathogens using statistical and artificial intelligence-based algorithms to analyse the differences in the Raman spectra. Silver nanocrystal substrates have been explored as a label-free method for the identification of food-borne pathogens *Escherichia coli*, *Salmonella typhymurium* and *Staphylococcus aureus* as well as differentiating live and dead bacteria.^127^ Ag as SERS substrates can differentiate between Gram-positive and Gram-negative bacteria with a characteristic peak at 497 cm^−1^ representing the cell wall polysaccharides of Gram-positive bacteria. Dipicolinic acid (DPA) has been used as a biomarker for the detection of bacterial spores, whilst Premasiri *et al* (2016) studied the different Raman spectra of bacteria at 785 nm wavelength and studied peaks related to adenine, hypoxanthine, xanthine, guanine, uric acid and adenosine monophosphate (AMP).^128^ Rodríguez-Lorenzo *et al.* (2019) reported that SERS-tagged gold nanostars with a monoclonal antibody were able to differentiate *Listeria monocytogenes* from *Listeria innocua* in real-time and in continuous flow under 100 seconds.^129^*Bacillus subtilis* produces an extracellular toxin subtilisin,^38^ which can also be considered as a biomarker for detecting *Bacillus subtilis.* However, there are only a few studies on detecting subtilisin using Raman spectroscopy in the literature. A more recent study by Li *et al.* (2020) used HfTe2-Au nanocomposites as label-free SERS substrates to detect *Escherichia coli*, *Listeria monocytogenes*, *Staphylococcus aureus* and *Salmonella* with the limit of detection of 10 CFU mL^−1^.^130^ Wu *et al.* (2013) previously reported the use of Ag nanorods for the identification of *Salmonella enteritidis*, *Escherichia coli* O157:H7 and *Staphylococcus epidermidis* in mung bean sprouts.^127^

Although label-free approaches offer a more powerful and straightforward analytical technique for detecting analytes, they also have salient limitations in demonstrating its effectiveness and translating it to real samples having complex biological matrixes, including the presence of salts, all types of cells, proteins and targets of interest. However, advancements in statistical algorithms and machine learning, *e.g.*, Banbury *et al.* (2019)^131^ and chemometrics techniques that require dimensionality reduction such as principal component analysis (PCA), and partial least squares have unlocked access to spectra deconvolution in complex matrixes. Therefore, capable of extracting information through complex data in an efficient manner has been a game changer for label-free SERS analysis in the food industry. A proof-of-concept for label-free and rapid detection of pathogens in food products has been demonstrated through direct detection of the surface of food. For instance, a study observed microbial levels in beef steaks stored in two different types of packaging (vacuum and modified atmosphere) at 4 °C, over 21 days. This was achieved by utilizing Raman spectroscopy in conjunction with partial least squares regression and comparing the results with traditional plating methods.^132^ In a related study, the progression of spoilage in chicken meat was analysed over time by integrating Fourier Transform Infrared (FTIR) spectroscopy and Raman spectroscopy. By combining Raman and FTIR spectra with the deconvolution of experimental bands into Lorentz components, these results successfully identified metabolic changes and spoilage progression over time. The findings included a noted reduction in protein levels (bands at 1655 and 1320 cm^−1^) and an increase in amino acids (band at 1675 cm^−1^), indicating spoilage within a 10-day period.^133^

**Fig. 5:**
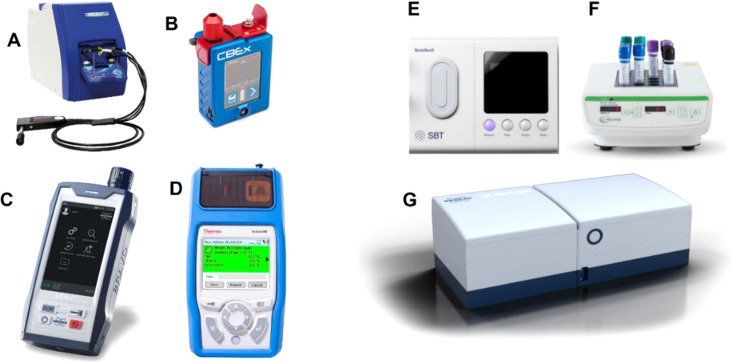
Commercially available portable Raman spectrometers. (A) i-Raman Plus 785H, Metrohm, (B) CBEx Raman spectrometer (Snowy Range, USA), (C) BRAVO, Bruker, (D) TruScan, Thermo Fisher Scientific, (E) Bactobox, SBT, (F) N-Light, Nemis Technologies and (G) Bactoscan, Foss. All the images have been obtained from the company's website.

Most of the current detection techniques are not applicable for PoN or PoC use *e.g.*, supermarkets, due to the highly specialised instrumental requirements, large size, lack of technical and chemical expertise and the overall time to results and maintenance cost. Thus, a portable device at PoC would enable real-time, rapid on-site detection^134^ and identification of pathogens. The capability of Raman spectroscopy to be deployed outside laboratory settings without significant loss of its performance has been yielding such emerging devices, including, for instance, Farber *et al.* (2018), who reported the use of a handheld Raman spectrometer coupled with chemometric analysis for the detection and identification of plant pathogens on maize kernels.^135^ Additionally, Egging *et al.* (2018) employed a hand-held Raman spectrometer for identifying and differentiating healthy and infected wheat and sorghum grains as a potential for early diagnosis of plant disease induced by fungal infections.^136^ Raman spectroscopy has also been widely used for quality control as a screening method for measuring freshness^137^ or detecting adulterants in food. Heuler *et al.* (2020) used First Guard, a Rigaku handheld 1064 nm Raman spectrometer as an *in situ* quality control sensor to detect chocolate bloom.^138^ Nekvapil *et al.* (2018) also employed the portable Raman systems (iRaman Plus, B & W TEK, 532 and 785 nm) (Fig. 5A) with fibre optic probes (BAC 102 Raman trigger) for freshness assessment in citrus fruits.^137^ Ellis *et al.* (2019) used a handheld CBEx Raman Spectrometer (Snowy Range, USA) (Fig. 5B) for the detection and quantification of methanol in spirit drinks.^139^ Karunathilaka *et al.* (2018) utilised handheld Raman spectrometer BRAVO by Bruker (Fig. 5C) and TruScan by Thermo Fisher Scientific (Fig. 5D) for the detection of melamine in powdered milk.^140^ However Nieuwoudt *et al.* (2016) utilised Raman spectroscopy to quantify N-rich compounds such as melamine, urea, ammonium sulphate, dicyandiamide and sucrose as adulterants in milk.^141^

### Industry 5.0: AI integration

Future challenges for addressing the trade-offs amongst sustainability, food security, and safety include adopting strategies to reduce wasted resources and resource footprints. In the face of climate change and biodiversity loss, accommodating ‘Green or Clean’ technology has attempted to prune the climate footprint and strengthen resilience to ensure food security and safety. Moreover, Green technology has promoted using environmentally sustainable and resilient design for manufacturing. As we are moving towards Industrial Revolution 5.0, we are witnessing a profound shift towards human-centric automation, where AI and advanced technologies collaborate with human creativity to achieve sustainable, personalised, and innovative solutions across the food industries. In line with this progression, Chen *et al.* (2023) proposed a novel risk prediction method called TabNet-GRA, which combines TabNet, a specialised deep learning architecture for tabular data, with Grey Relational Analysis (GRA) and an visualization system (FSRvis), aiding food safety supervision departments in decision-making.^142^

Paper-based substrates as green alternatives to SERS have emerged for the rapid identification and classification of bacteria and food contaminants. Paper-based substrates effectively increase the contact area between bacteria and the substrate, enhance the bacterial SERS signal, and allow on-site environmental monitoring and food analysis. Huang *et al.* (2019) developed a black phosphorus (BP)–Au filter paper based 3D substrate combined with chemometrics analysis such as principal component analysis and linear discriminant analysis (PCA–LDA) for low-cost, rapid, and accurate detection of foodborne bacteria (*Escherichia-coli*, *Listeria monocytogenes* and *Staphylococcus aureus*).^143^ Zhu *et al.* (2022) developed a self-assembled filter paper SERS substrate based on Au and Ag NPs for the identification of three common foodborne pathogenic bacteria *Staphylococcus aureus*, *Escherichia coli* and *Listeria monocytogenes*.^144^ Zhuang, J. *et al.* (2022) combined CRISPR/Cas12a and SERS to develop a recombinase polymerase amplification (RPA)-integrated microfluidic paper-based analytical device named “RPA-Cas12a-μPAD” for the detection of *Salmonella typhi* in milk and meat in 45 minutes.^145^ The detection limit was reported to be 3–4 CFU mL^−1^ for spiked milk and 1 to 10^8^ CFU mL^−1^ for meat samples. Chang *et al.* (2022) developed an aluminium-coated cellulose paper-based SERS (Al-PSERS) substrate for sensitive and rapid detection of food contaminants and coloured dyes such as Allura red and benzo[*a*]pyrene with detection limits of 3.5 and 0.15 ppm respectively, erythrosine and rhodamine B.^146^ In the light of industry 5.0, deep machine learning algorithms has been extensively employed for pathogen prediction in produce. Sharma *et al.* (2024) utilised a PCF sensor combined with PCA for feature extraction, Support Vector Machine (SVM) and Random Forrest (RF) classifier to predict the presence or absence of pathogen contamination.^147^ Similarly, Qiu *et al.* (2023) utilised SVM and deep learning architecture Convolutional Neural Network (CNN) for *in situ* detection of pathogens in chicken.^148^ Furthermore, Ma *et al.* (2023) developed a You Only Look Once version 4 (YOLOv4) algorithm integrated with phase-contrast microscopy to detect and quantify *Escherichia coli* microcolonies within 3 hours, achieving an average precision of 94%. The algorithm consists of CSP-darknet53-coco, a neural network used to extract microcolony features.^149^ However, Li *et al.* (2024) further proposed an improvised YOLOv7-based deep learning model for detection of poisonous snail species.^150^

## Conclusions and future perspectives

This review has highlighted a range of novel technologies, including Raman spectroscopy, nanotechnology-based biosensors, and nucleic acid-based assays, which hold promise for enhancing food safety and public health. For instance, the PCR-CRISPR-Fluorescence (PCF) based nucleic acid detection method proposed by Gao *et al.* (2021) demonstrated excellent sensitivity with a limit of detection (LoD) of 10 CFU mL^−1^ of *Salmonella* bacteria in just two hours. Despite its good specificity and lack of cross-reactivity, the technique's robustness remains a challenge, with rigorous sample preparation and a high level of technical expertise required, potentially limiting its applicability in non-specialized settings. Similarly, sensing techniques involving CRISPR systems, such as CRISPR-Cas13a and Cas12a, face stability issues under varying environmental and storage conditions, impacting their reliability and usability across different contexts.

Further advancements in biosensor technology, particularly in photoelectrochemical (PEC) and electrochemical biosensors, are promising, showing significant improvements in sensitivity, specificity, and portability. Future research should investigate the role of miR-21 and other microRNAs in the host response to foodborne pathogens, exploring their potential as indicators for such diseases. This could open new avenues for innovative sensing approaches like PEC. Additionally, given that many current techniques involve complex sample preparation steps, there is a need to explore methods that allow direct enumeration of microbial pathogens in complex food matrices.

The future of AI in this domain also offers significant potential, with algorithms becoming increasingly sophisticated and capable of managing complex tasks with enhanced accuracy and efficiency. A key focus for future studies will be on improving the interpretability and transparency of AI models, making them more accessible to non-experts. Moreover, integrating AI with emerging technologies such as quantum computing, blockchain, and the Internet of Things (IoT) could facilitate the resolution of more intricate problems and the enhancement of existing solutions. As AI's influence expands across various sectors, including healthcare, finance, and education, addressing ethical concerns and establishing robust regulatory frameworks will be crucial to balancing innovation with privacy, fairness, and accountability. As we move towards industry 5.0, the emphasis on human-centric automation and sustainability will further drive the evolution of AI, enabling more resilient, adaptive, and efficient solutions across various food industries for increasing food safety and security.

The expansion of AI applications is likely to increase human-AI collaboration, where AI augments rather than replaces human decision-making, necessitating research into optimising this synergy without undermining critical human skills. AI's potential contributions to sustainability also represent a promising area, particularly in addressing global challenges such as climate change and resource management. Finally, as AI technologies transcend borders, the development of international standards and governance structures will be essential to ensure equitable distribution of benefits and collective risk management.

In parallel, the COVID-19 pandemic has underscored the need for rapid, sustainable, and resilient technologies to address systemic gaps in the manufacturing, scalability, and distribution of existing technologies. Despite promising proof-of-concept studies, there remains a gap between fundamental research and the application of Surface-Enhanced Raman Spectroscopy (SERS) in the food industry, primarily due to batch-to-batch variation in SERS substrates. This challenge hampers SERS from competing with established technologies like fluorescence, but could be mitigated through the incorporation of standardization and calibration steps similar to those in commercial fluorescence kits.

From an instrumentation perspective, the development of portable and handheld devices for point-of-care (PoC) and point-of-need (PoN) testing is critical for enabling remote, in-field testing solutions. Handheld Raman devices, smartphones, and chip-based biosensors are emerging as researchers strive to replicate the capabilities of conventional analytical tools in powerful, miniaturized devices. Furthermore, the combination of label-free SERS with advanced machine learning and artificial intelligence could enable high-throughput reading and interpretation of complex data sets, such as those generated by Raman spectroscopy. Ultimately, the development of simple, reproducible, and reusable SERS substrates with high sensitivity, integrated with miniaturized Raman spectrometers, offers a sustainable and resilient solution for minimizing foodborne diseases and ensuring a safe food supply chain from farm to fork.

## Data availability

No primary research results, software or code have been included and no new data were generated or analysed as part of this review.

## Conflicts of interest

There are no conflicts to declare.
